# Zebrafish teeth as a model for repetitive epithelial morphogenesis: dynamics of E-cadherin expression

**DOI:** 10.1186/1471-213X-10-58

**Published:** 2010-06-01

**Authors:** Barbara Verstraeten, Ellen Sanders, Jolanda van Hengel, Ann Huysseune

**Affiliations:** 1 Evolutionary Developmental Biology, Ghent University, Belgium; 2 Molecular Cell Biology Unit, Department for Molecular Biomedical Research, VIB Ghent, Belgium; 3 Department of Biomedical Molecular Biology, Ghent University, Ghent, Belgium

## Abstract

**Background:**

The development of teeth is the result of interactions between competent mesenchyme and epithelium, both of which undergo extensive morphogenesis. The importance of cell adhesion molecules in morphogenesis has long been acknowledged but remarkably few studies have focused on the distribution and function of these molecules in tooth development.

**Results:**

We analyzed the expression pattern of an important epithelial cadherin, E-cadherin, during the formation of first-generation teeth as well as replacement teeth in the zebrafish, using *in situ *hybridization and whole mount immunostaining to reveal mRNA expression and protein distribution. E-cadherin was detected in every layer of the enamel organ during the different stages of tooth development, but there were slight differences between first-generation and replacement teeth in the strength and distribution of the signal. The dental papilla, which is derived from the mesenchyme, did not show any expression. Remarkably, the crypts surrounding the functional teeth showed an uneven distribution of E-cadherin throughout the pharyngeal region.

**Conclusions:**

The slight differences between E-cadherin expression in zebrafish teeth and developing mouse and human teeth are discussed in the light of fundamental differences in structural and developmental features of the dentition between zebrafish and mammals. Importantly, the uninterrupted expression of E-cadherin indicates that down-regulation of E-cadherin is not required for formation of an epithelial tooth bud. Further research is needed to understand the role of other cell adhesion systems during the development of teeth and the formation of replacement teeth.

## Background

The dentition of vertebrates consists of repetitive units, teeth, which have an epithelial as well as a mesenchymal component. Some features of tooth development are shared (e.g. formation of an epithelial bud, condensation of mesenchyme, polarized deposition of mineralizing matrices) whereas others are unique to certain taxa (e.g. different ways of attachment, different ways of eruption, whether enamel or enameloid is produced) [1-3].

Like many other vertebrate organs, teeth arise by epithelio-mesenchymal interactions. Their development starts in the same way in all vertebrates by the formation of an epithelial thickening (placode) that invaginates into the underlying mesenchyme and forms a bud surrounding the condensed mesenchyme (dental papilla) [2].

The zebrafish, a widely used model in genetic, molecular and developmental research, has no oral teeth, but teeth attached to the fifth branchial arch only [3]. The complete dentition of the zebrafish consists of three rows of teeth on each side, all of which are replaced throughout life: a ventral row (V) of five teeth, a mediodorsal row (MD) of four teeth and a dorsal row (D) of two teeth. The teeth in the ventral row are named 1V to 5V, rostral to caudal [4]. The first tooth bud starts to develop after 2 dpf (days post-fertilization) at position 4V and is quickly followed by the development of the tooth germs at positions 3V and 5V. The teeth in the two rostral positions, 2V and 1V, develop at 12 and 16 dpf, respectively [4]. Replacement of zebrafish first-generation teeth starts early, between 3 and 4 dpf for the first tooth (position 4V). In the zebrafish, buds of first-generation teeth develop directly from the pharyngeal epithelium. In contrast, buds that will form replacement teeth develop from an epithelial outgrowth at the base of the epithelial crypt surrounding the tip of the erupted functional tooth. This outgrowth is called the successional lamina [5]. Formation of the successional lamina and of the epithelial tooth bud, as well as condensation of the mesenchyme to form the dental papilla, requires substantial rearrangements of cells. It is very likely that cell adhesion molecules play an important role in these rearrangements. Although the importance of cell-cell adhesion during morphogenesis has been known for quite some time [6], there are remarkably few studies on the distribution and function of cell adhesion molecules in tooth development [7-10]. This poor knowledge stands in sharp contrast with the amount of data collected over the previous years on regulatory mechanisms in tooth development, which involves transcription factors, growth factors, signaling molecules and receptors of the extracellular matrix [11].

The few studies about cell adhesion molecules in tooth development have focused on mouse or human teeth or on one type of cell only [7,9,10]. Moreover, the focus has been mainly on embryonic tooth development (formation of first-generation teeth) and little or no attention has been paid to the process that underlies the renewal of teeth (development of replacement teeth).

Cadherins constitute a large family of Ca^2+^-dependent adhesion molecules. The family is divided into several subfamilies, of which the classical cadherins are the most studied [6]. E-cadherin belongs to the subfamily of classical cadherins and is an epithelial cadherin responsible for the maintenance of epithelial cell layers [12]. Other classical cadherins are typical for different tissues: e.g. N-cadherin is expressed in muscle and neural tissue, R-cadherin in forebrain and bone, and VE-cadherin in endothelial cells [13].

Cadherins play important roles in cell adhesion through their link to the actin cytoskeleton. Newly synthesized cadherins are transported to the cell membrane while coupled to β-catenin. At the cell membrane p120 catenin binds to the juxtamembrane domain of the intracellular part of the cadherin, thus stabilizing it at the membrane [14]. β-catenin and plakoglobin compete for binding to the C-terminal domain of E-cadherin, and are each also linked to α-catenin. The latter can also bind the actin cytoskeleton. Together, these molecules form the cadherin-catenin complex. When the expression of E-cadherin is downregulated (e.g., in tumor development), cell adhesion decreases and cell migration increases [12,15,16]. Whereas different intracellular signaling pathways can modulate the formation of the complex and the strength of the adhesion, cadherin-mediated adhesion itself can also influence intracellular signaling via Rho-GTPases or indirectly via several growth factors [17,18]. Cadherin-dependent signaling influences cellular processes such as proliferation, survival, differentiation, morphogenesis and migration. These processes are important during embryogenesis and organogenesis [19-21].

Jamora et al. (2003), using mouse hair follicle as a model, proposed that E-cadherin is downregulated at the start of the formation of an epithelial bud as a result of activation of the Wnt-signaling pathway, concomitantly with an inhibition of BMP-signaling. Such down-regulation would allow rearrangement of the epithelial cells to enable formation of a bud, such as during the formation of glands or hair follicles.

Our study was inspired by the lack of data on the distribution and role of E-cadherin during the formation of tooth buds and by the hypothesized role of E-cadherin down-regulation in epithelial bud formation. We report on the dynamics of E-cadherin expression, both at the mRNA and protein level, in first-generation teeth and their successors in an animal displaying continuous tooth renewal, the zebrafish. This is part of a larger study aiming at elucidating the role of specific cell adhesion molecules in the development and renewal of teeth.

## Results

E-cadherin mRNA expression and protein localization coincided at all material examined. Therefore, we do not distinguish between them in the following sections. Outside the prospective dental placode E-cadherin expression was also detected in the pharyngeal epithelium as well as in the presumptive gill slits. E-cadherin was detected in the pharyngeal epithelium, both before (48 hours post-fertilization (hpf)) and after (56 hpf) the formation of the pharyngeal cavity. All layers of the pharyngeal epithelium expressed E-cadherin to the same extent in 40 hpf until 6 days post-fertilization (dpf) old fish. Also the epithelial lining of the presumptive gill slits and the cells within the multiple layers of the presumptive keratinized pad, facing the developing pharyngeal jaws, were E-cadherin-positive. However, at about 4 dpf, the most apical layer of the keratinized pad, i.e. the layer that is shed, lost its E-cadherin expression.

### Distribution of E-cadherin during the development of first-generation teeth

Below we will describe the expression of E-cadherin in tooth 4V^1^, the first tooth to develop, as representative of the pattern observed in first-generation teeth. We found no difference in expression pattern or cellular distribution of E-cadherin during development when we compared teeth 3V^1 ^and 5V^1 ^with 4V^1^.

#### Initiation and morphogenesis

Initiation of tooth development is characterized morphologically by formation of an epithelial thickening, the placode. In this area, the cells of the basal layer of the epithelium are columnar and polarized. These cells start to invaginate into the underlying mesenchyme during morphogenesis.

**Figure 1:**
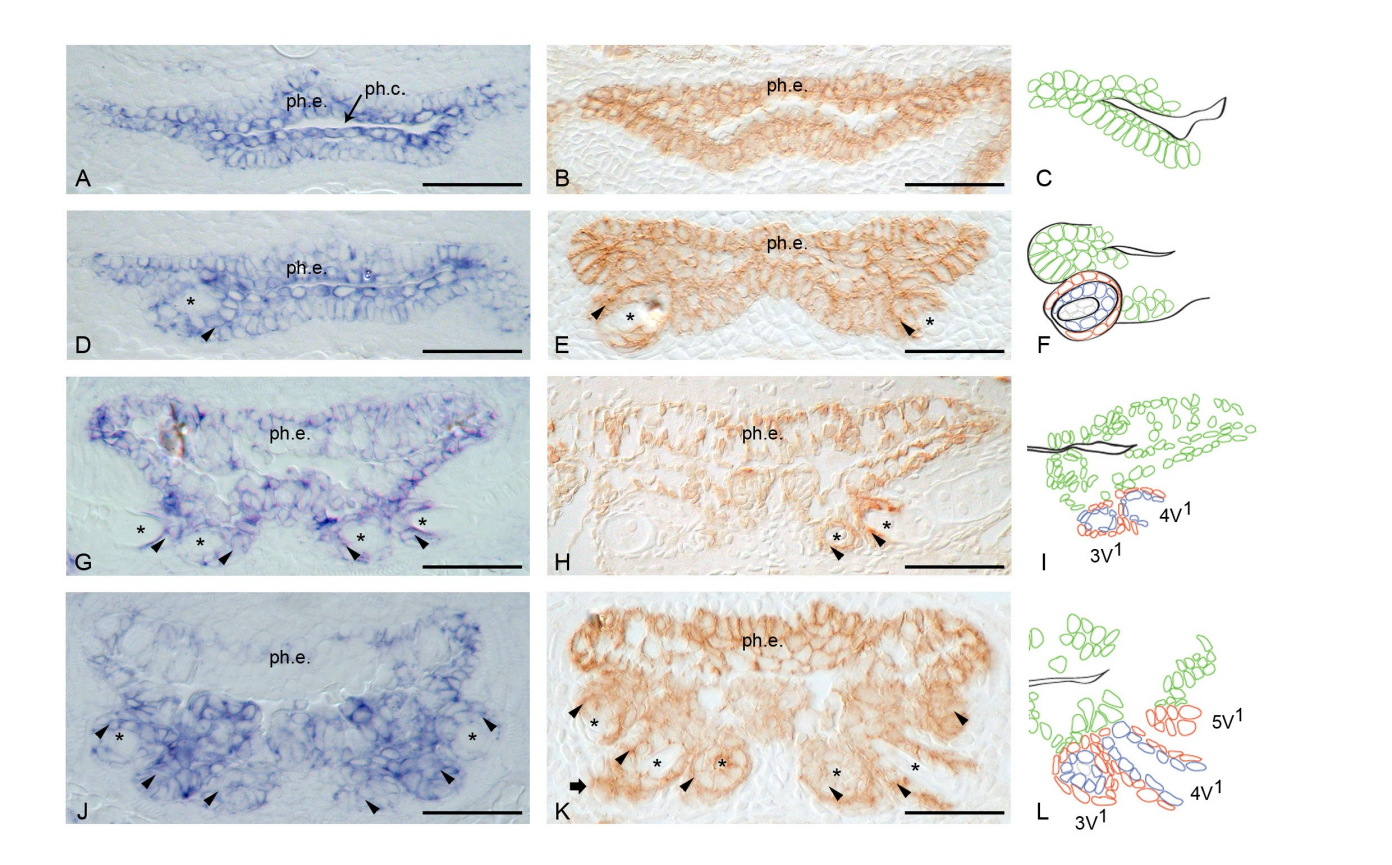
**Expression pattern of E-cadherin in first-generation teeth**. Left panels: mRNA expression; middle panels: protein expression; right panels: diagram of different cell layers of the tooth. A, B, C: Initiation phase. D, E, F: Morphogenesis phase; G, H, I: Early cytodifferentiation and attachment phase; J, K, L: Late cytodifferentiation phase and initiation of successor. ph.c.: pharyngeal cavity; ph.e.: pharyneal epithelium; *: dental papilla; arrowhead: enamel organ; block arrow: initiation replacement tooth. Diagrams: green cells: pharyngeal epithelium; red cells: outer dental epithelium; blue cells: inner dental epithelium. Scale bars = 2 5 μm.

Cells that form the placode show strong expression of E-cadherin. No difference in strength of the signal could be detected between placodal cells and the non-differentiated parts of the pharyngeal epithelium (Figure 1A, B, C; Table 1).

**Table 1:** Overview of expression of E-cadherin in the different cell layers of a first-generation tooth on the basis of mRNA and protein distribution

	epithelium		mesenchyme	
	ide	ode	odontoblasts	dental papilla
**initiation**	++	++	-	-
**morphogenesis**	++	++	-	-
**early cytodifferentiation**	++	++	-	-
**late cytodifferentiation**	++	++	-	-
**attachment**	++	++	-	-

The epithelial part of the tooth remains positive for E-cadherin throughout all stages of tooth development. Odontoblasts and dental papilla show no E-cadherin expression at any developmental stage.

During morphogenesis the epithelium starts to protrude into the mesenchymal layer underneath. Subsequently, the epithelial cells start to differentiate into two layers of cells: the inner dental epithelium (IDE) and the outer dental epithelium (ODE). These two layers together form the enamel organ of the developing tooth. Opposite the epithelial cells, mesenchymal cells condense to form the dental papilla of the tooth. During morphogenesis stage E-cadherin was expressed in the cells of both the IDE and the ODE, but no signal was present in the mesenchymal part of the tooth, the dental papilla (Figure 1D, E, F; Table 1).

#### Early cytodifferentiation

While the free margin of the enamel organ, called the cervical loop, grows deeper into the mesenchyme, the IDE cells differentiate into ameloblasts and the cells of the dental papilla into odontoblasts. The ameloblasts start to produce enameloid. The odontoblasts, on the other hand, start to produce predentin which will later mineralize into dentine.

During early cytodifferentiation, the ODE and ameloblasts maintain strong expression of E-cadherin, at both the mRNA and the protein level. Again, all the cells that form the dental papilla show no sign of expression of this cell adhesion molecule (Fig. 1G, H, I, tooth 3V^1^; Table 1).

#### Late cytodifferentiation

As cytodifferentiation proceeds, the matrix becomes clearly visible on the sections, but enameloid and dentine are hardly distinguishable. The cervical loop is now lying close to the cartilage of the branchial arch but no attachment bone has been deposited yet.

During late cytodifferentiation stage, the expression pattern has not changed. Both the ODE and ameloblasts remain positive while the odontoblasts remain negative for E-cadherin (Fig. 1J, K, L; Table 1).

#### Attachment and eruption

Finally, the attachment bone is formed in the prolongation of the tooth base and the tooth is attached to the perichondral bone surrounding the cartilaginous branchial arch. The epithelial layers overlying the tooth tip then fold back, thus exposing the tooth tip into the pharyngeal lumen [22]. The tooth is now erupted and becomes functional.

The expression pattern of E-cadherin in a functional tooth is identical to that in a developing tooth. Both epithelial layers, ODE and IDE, which form the reduced enamel organ at this stage, are strongly positive for both E-cadherin mRNA and protein. This is in sharp contrast to the dental papilla, which does not express E-cadherin at all (Fig. 1H, tooth 4V^1^; Table 1).

### Distribution of E-cadherin during the development of replacement teeth

**Figure 2:**
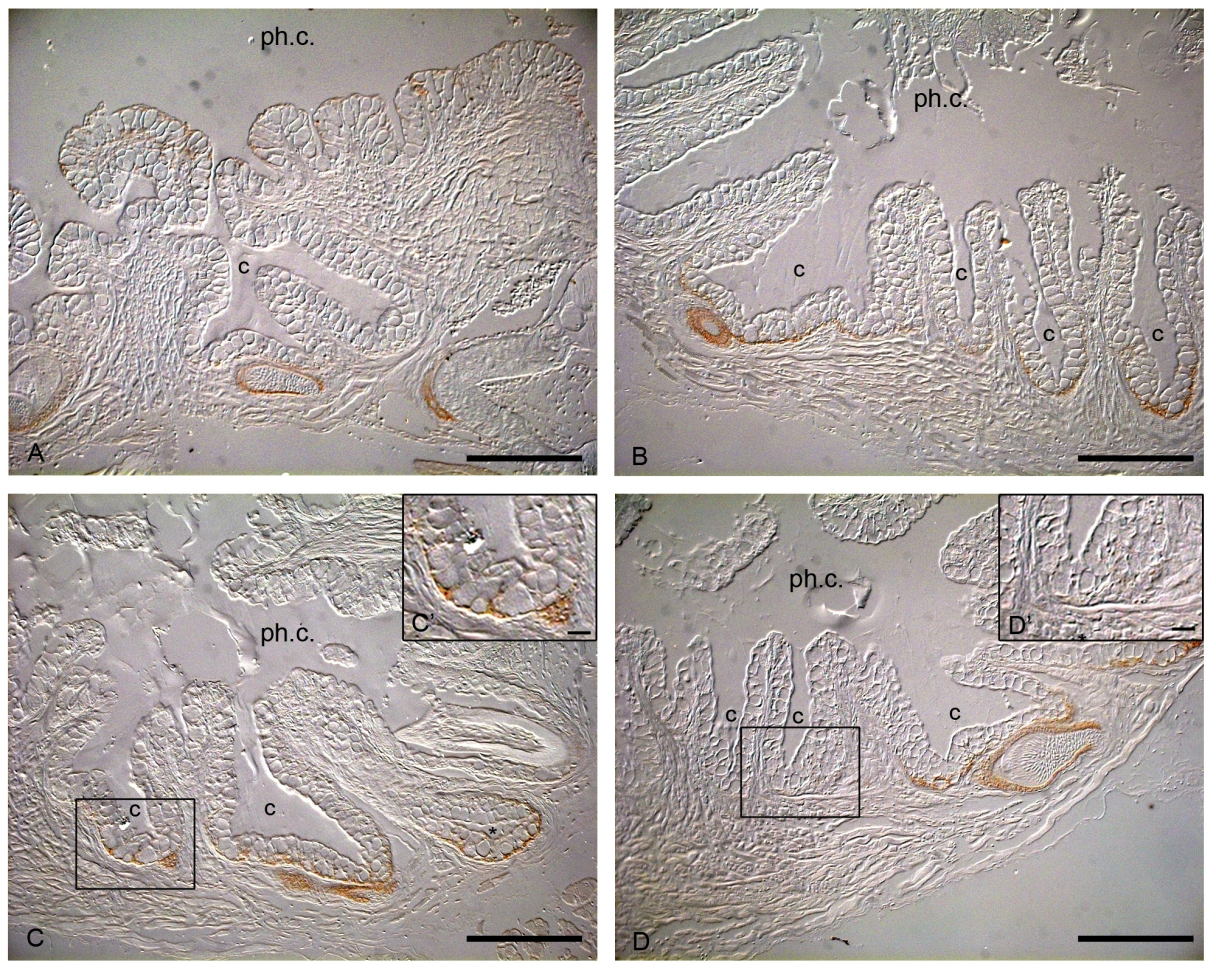
**Uneven distribution of E-cadherin in the crypt epithelium**. Sections of whole mount immuocytochemical examination of dissected adult jaw. The rostral crypts in the pharyngeal region of adult zebrafish express E-cadherin throughout the epithelium (A). This contrasts to the more caudal crypt epithelium, which loses E-cadherin expression, except along the crypt bases (B). Cells placed rostrally at the base of the crypt always have stronger E-cadherin expression (C, C') than cells placed more caudally within the same crypt (D, D'). c: crypt; ph.c.: pharyngeal cavity. Scale bars = 50 um, scale bar C', D' = 10 μm.

To analyze E-cadherin distribution during tooth replacement, whole mount immunohistochemistry was performed on specimens of different ontogenetic stages up to 6 dpf and on dissected jaws of an adult zebrafish. Additionally, *in situ *hybridization was conducted on specimens of similar stages and on dissected jaws of an adult fish to clarify the expression pattern of this cell adhesion molecule. The examination of adult jaws provided better insight into the mRNA expression and distribution of E-cadherin protein in teeth of later generations. In adults, all eleven tooth positions are established and replacement teeth are in different stages of development. Moreover, the teeth are larger than first-generation teeth and thus the tissues consist of many more cells. This enables detailed observations of the expression at the cellular level. In the description below, we focus on the results obtained from the analysis of whole mount immunostaining of dissected adult jaws. The branchial arches express E-cadherin in the epithelial lining of the gills and of the pharyngeal cavity. We observed expression in the rostral part of the keratinized pad opposite the pharyngeal jaws, but not in the caudal part. An E-cadherin signal is evident in the crypt epithelium surrounding the tip of each functional tooth, but it is distributed unevenly. The epithelium of the rostral crypts consists of cells all of which express E-cadherin (Fig. 2A). The E-cadherin signal decreases in crypts more caudally, and finally disappears in the most caudal crypts, except at their base (Fig. 2B). In each crypt belonging to a specific functional tooth, whether positioned more rostral or more caudal, the rostral cells at the base of the crypt always have stronger E-cadherin expression than the cells that constitute the caudal side of the same crypt (Fig. 2C, C', D, D').

We observed a dynamic pattern of E-cadherin expression during the formation of a replacement tooth early in post-embryonic development and in adults.

The stages of the odontogenesis distinguished during development of replacement teeth resemble those described for first-generation teeth.

#### Initiation

**Figure 3:**
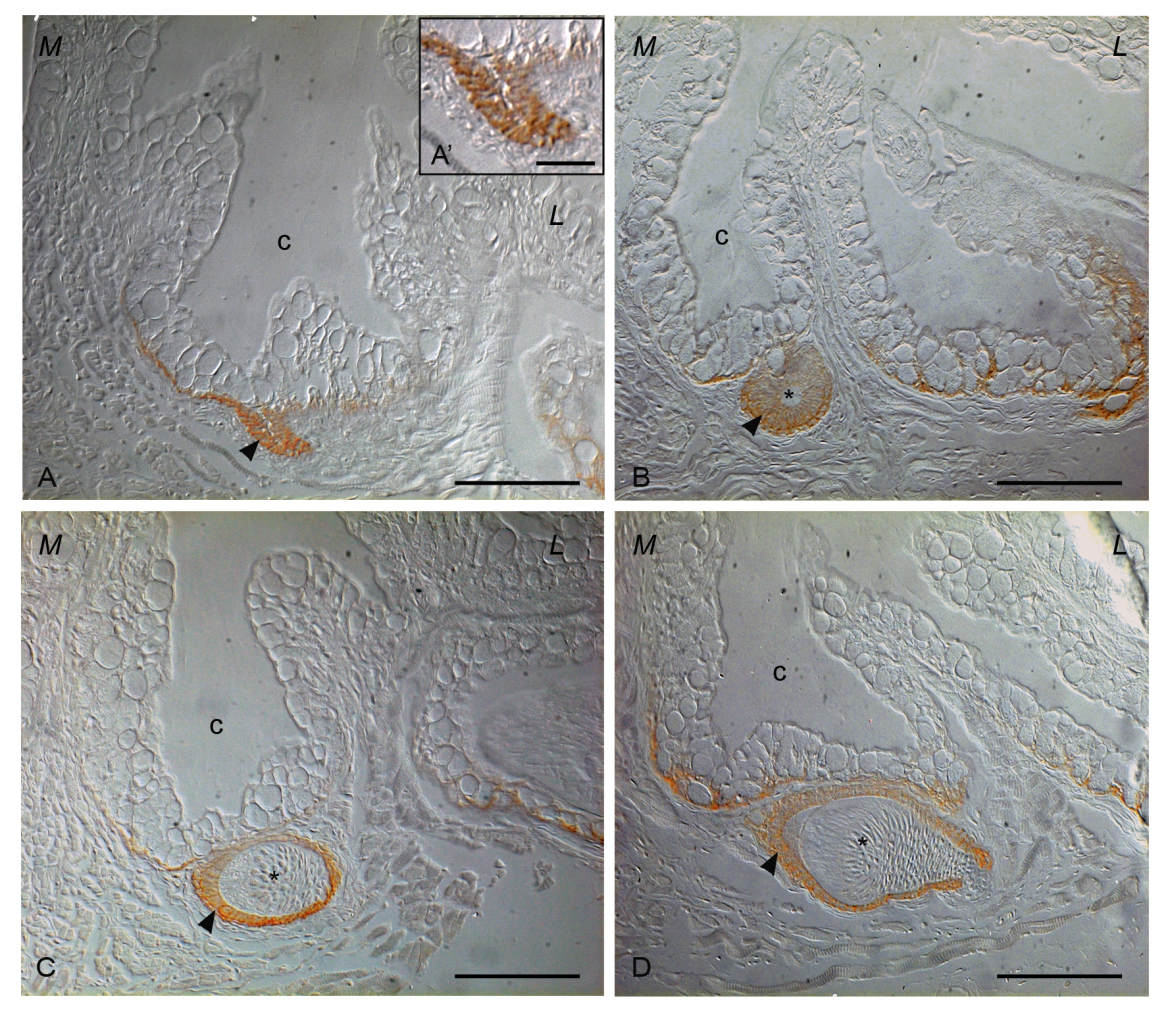
**E-cadherin expression pattern during tooth replacement**. Sections of whole mount immunocytochemical examination of dissected adult jaw. A, A': Successional dental lamina is positive for E-cadherin; B: Morphogenesis phase; C: Early cytodifferentiation, IDE and ODE express E-cadherin; D: Late cytodifferentiation. *: dental papilla; arrowhead: enamel organ; c: crypt slightly posterior to the tip of the functional predecessor; L: lateral; M: medial. Scale bars = 50 μm, scale bar A' = 25 μm.

Unlike first-generation teeth, replacement teeth do not arise directly from the pharyngeal epithelium. They are formed medial to their predecessors from an epithelial protrusion, called the successional lamina (Fig. 3A), which develops close to the base of the crypt surrounding the tip of the predecessor. During the first replacement cycles the successional lamina is inconspicuous because the teeth are small. It becomes prominent in later ontogenetic stages, when teeth are larger and have gone through several replacement cycles [5]. As replacement teeth develop, they assume a horizontal position, with their dorsal side closely adjoining the crypt epithelium (Fig. 3B-D).

The successional lamina expresses E-cadherin, except for the centre of the lamina (Fig. 3A'). This pattern was observed consistently for all four replacement teeth in the ventral row observed in this developmental stage. The signal in the cells of the successional lamina is extremely strong. During this stage corresponding to the initiation stage of the replacement tooth, the base of the crypt of the functional tooth expresses E-cadherin as well, particularly on the medial side of the crypt (Fig. 3A; Table 2).

**Table 2:** Overview of expression of E-cadherin in the different cell layers of a replacement tooth

	epithelium		mesenchyme	
	ide	ode	odontoblasts	dental papilla
**initiation**	++	++	-	-
**morphogenesis**	++	++	-	-
**early cytodifferentiation**	++ *	++ *	-	-
**late cytodifferentiation**	++ *	++ *	-	-
**attachment**				

The two layers of the enamel organ, IDE and ODE, maintain the expression of E-cadherin. The mesenchyme-derived dental papilla does not express this cell adhesion molecule during development. *: stronger expression of E-cadherin on the ventral side of the developing tooth.

**Figure 4:**
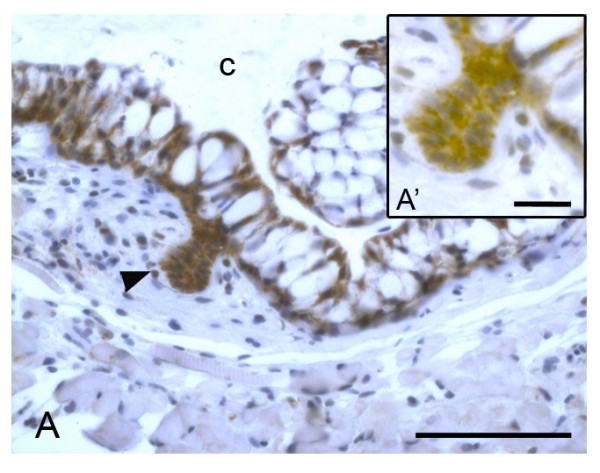
**Beta-catenin immunolocalization in the successional lamina**. The onset of formation of a replacement tooth, the successional lamina, has strong p-catenin expression along the membrane of every cell (A, A'). This β-catenin expression co-localizes with the expression of E-cadherin suggesting that the cadherin-catenin complex is functional as adhesive force. arrowhead: successional lamina; c: crypt surrounding the tip of the functional predecessor. Scale bar = 50 μm, scale bar A' = 15 μm.

It should be noted that the successional lamina displays strong, membrane-bound, immunolocalization of β-catenin (unpublished results and Fig. 4).

#### Morphogenesis

As the distal part of the successional lamina develops into the enamel organ of the new tooth bud, a strong E-cadherin signal is maintained. Likewise, the base of the crypt maintains the same expression pattern seen in the initiation phase, i.e. the medial side of the crypt is more intensely stained than the lateral side (Fig. 3B; Table 2).

#### Early cytodifferentiation

The cells of the enamel organ of the new tooth, while growing obliquely into the underlying mesenchyme, differentiate into the IDE and ODE, which maintain E-cadherin expression. E-cadherin expression is remarkably stronger on the ventral side of the developing tooth than on its dorsal side. At the same time, the layer of mesenchymal cells opposite the IDE differentiates into odontoblasts. The mesenchymal cells express no E-cadherin (Fig. 3C; Table 2).

From this stage onwards, the strength of the E-cadherin signal in the different cell layers of the teeth decreases. Moreover, its expression at the base of the crypt also diminishes. Yet, the medial side of the crypt still expresses E-cadherin more strongly than the lateral side.

#### Late cytodifferentiation

Late cytodifferentiation is marked by the presence of a noticeable amount of matrix (enameloid first, followed by dentine). During this stage, the IDE and ODE continue to express E-cadherin, albeit at a lower level, while the dental papilla remains negative (Fig. 3D; Table 2). The strongest expression is in the cells on the ventral side of the tooth. In the crypt of the corresponding functional tooth, the expression of E-cadherin becomes barely noticeable.

## Discussion

We examined mRNA expression and protein localization of the cell adhesion molecule E-cadherin during the development stages of first-generation teeth and replacement teeth in zebrafish. We observed no difference in the localization of E-cadherin transcripts or protein, which indicates that transcription leads to translation. In both first-generation and replacement teeth, the two layers that constitute the enamel organ express E-cadherin during the different stages of tooth formation. The dental papilla, on the other hand, is consistently negative for E-cadherin. Despite this overall pattern, there are some differences in the distribution of the E-cadherin signal between first-generation and replacement teeth. First, unlike first-generation teeth, developing replacement teeth display a decrease of E-cadherin expression beyond morphogenesis stage. Likewise, the crypt surrounding the tip of the functional tooth maintains E-cadherin expression along the medial side of the crypt base longer than along the lateral side. This regional difference could be related to the position of the developing replacement tooth with respect to the crypt base. The cells at the base of the crypts have E-cadherin expression at the position from which the successor arises from the outer dental epithelium of the predecessor.

Remarkably, crypts displaying an E-cadherin signal were unevenly distributed throughout the pharyngeal region. Anteriorly, all crypts showed a signal, whilst the signal diminished more posteriorly within the pharyngeal cavity, until no expression was left in the most posterior crypts, except at their base. An even more puzzling finding was that only the crypts of the ventral teeth seem to express E-cadherin. No expression was found at the base of the crypts of the medio-dorsal or dorsal teeth. We cannot exclude the possibility that a cadherin-switch could be responsible for this differential expression pattern as described during morphogenesis and tumor progression [23,24].

Previous studies on cell adhesion molecules in teeth focused on mouse and human teeth [7,10]. Moreover, these studies examined the expression pattern during development of first-generation teeth or focused on specific cell types [9]. During the development of teeth in the mouse, E-cadherin is expressed in all epithelial cells during the initiation phase. Distinct E-cadherin expression is also observed during morphogenesis in the dental lamina, stellate reticulum, stratum intermedium, ODE and the cervical loop in mouse tooth development. During cytodifferentiation, the polarizing ameloblasts are also positive. The expression pattern of E-cadherin in mouse and zebrafish teeth is similar, the major difference being the absence of E-cadherin expression in the IDE during morphogenesis in the mouse. The patterns of E-cadherin and P-cadherin expression in IDE cells in the mouse change during development. During morphogenesis of the developing mouse molar, the IDE expresses P-cadherin but not E-cadherin [9]. Expression of P-cadherin has been associated with the proliferation of the cells and with their ability to induce condensation of the surrounding mesenchyme [10]. Moreover, the differential expression of E- and P-cadherin in IDE cells is also related to their differentiation stage. Undifferentiated IDE cells are positive for P-cadherin, whereas polarized and differentiated ameloblasts in mouse express E-cadherin [9]. Heymann et al. (2002) did not detect any expression of E-cadherin during the initiation phase of human deciduous teeth but observed that the amount of protein in the enamel organ increased during morphogenesis. During later developmental stages, E-cadherin expression diminishes in the apical to coronal direction, while N-cadherin is up-regulated. This expression pattern is explained by the suggestion that increased N-cadherin expression might be important for ameloblast transformation and polarization required for enamel matrix secretion [7].

It is important to keep in mind several features that distinguish zebrafish from mammalian (mouse and human) teeth. First, the mouse is monophyodont and all studies on murine tooth development therefore concern first-generation teeth. Second, the structure of the enamel organ differs considerably from that of zebrafish. The enamel organ of zebrafish teeth possesses no stellate reticulum or stratum intermedium, and IDE and ODE are directly apposed. Ameloblasts in mammalian teeth are involved in deposition of enamel matrix, its subsequent mineralization and removal of organic matrix during enamel maturation; therefore, ameloblasts cycle through multiple functional stages [25,26]. In contrast, ameloblasts in zebrafish are involved in the deposition of enameloid, which is both structurally and developmentally different from mammalian enamel [2,27,28]. Moreover, tooth tissues of zebrafish consist of substantially fewer cells than their murine counterparts. All these factors could contribute to the slight differences in E-cadherin expression pattern between zebrafish and mouse teeth.

Jamora et al. (2003), using data from the mouse, hypothesized that formation of an epithelial bud is facilitated by down-regulation of E-cadherin in response to Wnt-signaling. Consistent with their hypothesis, they found that over-expression of E-cadherin inhibits invagination and production of hair follicles in mouse skin [29]. Morphogenesis of a tooth bud and a hair bud share many features [11,30,31]. Thus, we focused specifically on whether we can observe down-regulation of E-cadherin during formation of tooth buds of first-generation or replacement teeth. Contrary to what could be predicted based on the suggestion made by Jamora et al. (2003), we observed uninterrupted and very strong expression of E-cadherin during formation of the placode (in first-generation teeth) or the successional lamina (in replacement teeth) [32,33]). E-cadherin mutants of zebrafish, as well as morphants (induced by morpholino injections) do not survive gastrulation [34,35]. As teeth start to develop at 2 dpf, loss-of-function experiments to unravel the function of E-cadherin during tooth development were therefore not possible to conduct.

Many tissues (not just hair follicles, but also glands such as mammary and salivary glands) share the same developmental dynamics of invagination of an epithelial layer into the underlying mesenchyme to form an epithelial bud. Studies describing the regulation of different cell adhesion molecules during this process in hair follicles, mammary glands and salivary (submandibular) glands show that expression of different cell adhesion systems is dynamic early during morphogenesis. In these tissues, desmosomal and hemidesmosomal components are downregulated during formation of a placode and there is complete loss of these structures in the epithelial bud [36-39].

Recent studies on cancer and tumor development consider the possibility that E-cadherin can remain at the cell membrane, yet not function any longer in adhesion. This contrasts to the long standing idea that disruption of the cadherin-catenin complex would lead to internalization and degradation of E-cadherin. Rather, it has been suggested that it might be the loss of β-catenin that makes the cadherin-catenin complex incompetent, thereby reducing adhesion strength [40,41]. This emerging hypothesis can explain how a straight layer of cells can change cell shape and position without loss of E-cadherin expression. The distinct membrane-bound presence of β-catenin in the successional lamina of zebrafish replacement teeth, however, argues against this possibility.

In conclusion, our present data, together with data published on other organ systems, lead us to suggest that formation of an epithelial bud is not necessarily associated with down-regulation of E-cadherin. Other cell adhesion systems can play an important role in the first stages of development in tissues that arise through epithelio-mesenchymal interactions.

## Conclusions

In this study, we present the expression pattern and protein distribution of the epithelial cell adhesion molecule E-cadherin during the different stages of tooth development in the zebrafish. We have shown that E-cadherin is present in the epithelial derived part of the developing tooth, both in the inner as well as in the outer dental epithelium. The mesenchymal derived dental papilla remains negative for E-cadherin throughout development. The same expression pattern is found in replacement teeth.

Zebrafish replace their teeth continuously throughout life and therefore their teeth represent an attractive model for studying repetitively renewing structures that start their formation with an epithelial bud. Our data do not show a down-regulation of E-cadherin at the onset of formation of an epithelial bud such as proposed in hair follicles by Jamora et al. (2003).

Further research is needed to understand the role of other cell adhesion systems during the development of teeth and the formation of replacement teeth.

## Methods

### Fish strains

Zebrafish *(Danio rerio) *were mated and eggs were allowed to develop at 28.5°C in a 10-h dark/14-h light cycle. The embryos were sacrificed according to the Belgian law on the protection of laboratory animals (KB d.d. 13 September 2004) by an overdose of MS222 (3-aminobenzoic acid ethyl ester) at specific times. The first embryos were sacrificed at 40 hpf and then after every 4 hours until 4 dpf. From 4 dpf until 6 dpf, fish were sacrificed every 8 hours.

Fifth branchial arches were dissected from adult zebrafish under a Leica MZ Apo dissecting microscope using microscissors.

### Tissue processing

The embryos, larvae and dissected fifth branchial arches were fixed overnight at 4°C in 4% paraformaldehyde (PFA). Before storing the embryos and larvae in methanol (MeOH), they were depigmented in 0.5% KOH and 0.05% H_2_O_2 _in phosphate buffered saline (PBS) for 30 minutes to 1 hour at room temperature (RT). They were washed twice with 1× PBS put through several MeOH steps (25%, 50%, 75%) and stored at -20°C in 100% MeOH. The dissected branchial arches were not depigmented but, immediately after fixation, they were put through a series of MeOH and stored in 100% MeOH at -20°C.

Adult PFA-fixed jaws selected for immunolocalization of β-catenin were paraffin-embedded according to routine procedures and serially sectioned at 5 μm.

### Immunohistochemistry

The embryos and larvae selected for whole mount immunohistochemistry were rehydrated through a descending MeOH series. They were next treated with proteinase K for 40 minutes in a concentration depending on the age of the animal. This pretreatment with proteinase K does not affect the antigens as evidenced by strong positive staining of typical epithelial tissues. The primary antibody used was the E-cadherin antibody clone 36 against the C-terminal domain of human E-cadherin, BD Transduction Laboratories, tested for specificity on a western blot using a zebrafish lysate. This antibody was successfully applied to zebrafish [42,43]. The specimens were incubated with the primary antibody overnight at 4°C (dilution 1:300 in blocking solution) and incubated for 20-24 h at 4°C with the secondary antibody (Polyclonal goat anti-mouse immunoglobulin biotinylated, Dako, diluted 1:300 in blocking solution). They were next treated with the streptABComplex-horseradish peroxidase (Dako) and the location of the antibodies was detected with DAB (Biogenex). Control embryos were treated with secondary antibody only. To analyze the signal in detail, the specimens were subsequently dehydrated, embedded in epon, serially sectioned at 4 μm, and the sections were mounted with Depex.

Immunolocalization on paraffin sections was performed according to standard procedures [44] using β-catenin primary antibody (C7207, Sigma, dilution 1:1000) and polyclonal goat anti-mouse immunoglobulin biotin-coupled secondary antibody (Dako, dilution 1:500).

### Probe synthesis and *in situ *hybridization

Digoxigenin-labeled probes were synthesized from template cDNA of *cdh1 *clone 26 in plasmid BlueScript (plasmid kindly provided by Dr. James Marrs, Indiana University Medical Center, IN). The plasmid was linearized with Xhol and transcribed with T3 RNA polymerase to generate the sense probe and linearized with *Eco*RI and transcribed with T7 RNA polymerase to obtain the anti-sense probe.

*In situ *hybridization with sense and antisense probes was performed following the protocol established by Pamela Raymond (University of Michigan, Ml) http://www.mcdb.lsa.umich.edu/labs/praymond. The probes were detected with NBT/BCIP. After staining, the specimens were fixed overnight at 4°C in 4% PFA. They were next dehydrated, embedded in epon, and serially sectioned at 4 μm. The sections were mounted with Depex.

All sections were examined under a Zeiss Axio Imager Microscope equipped for DIC and photographed using an Axiocam MRC videocamera.

## Authors' contributions

BV contributed to conception and design of the project, the acquisition of all data, she analysed the data and was involved in drafting the manuscript. ES significantly contributed to the establishment of ISH protocols. JVH contributed to design, extensively discussed results and revised the manuscript for intellectual content. AH contributed to conception of the project, interpreted results, and helped to draft the manuscript. All authors read and approved the final manuscript.
